# A Color-Picture Version of Boston Naming Test Outperformed the Black-and-White Version in Discriminating Amnestic Mild Cognitive Impairment and Mild Alzheimer's Disease

**DOI:** 10.3389/fneur.2022.884460

**Published:** 2022-04-25

**Authors:** Dan Li, Yue-Yi Yu, Nan Hu, Min Zhang, Li Liu, Li-Mei Fan, Shi-Shuang Ruan, Fen Wang

**Affiliations:** ^1^Innovation Center for Neurological Disorders, Department of Neurology, Xuanwu Hospital, Capital Medical University, Beijing, China; ^2^Discipline of Pediatrics & Child Health, School of Clinical Medicine, UNSW Medicine & Health, UNSW Sydney, Sydney, NSW, Australia; ^3^Department of Radiology, Xuanwu Hospital, Capital Medical University, Beijing, China; ^4^Department of Neurology, Xuanwu Hospital, Capital Medical University, Beijing, China

**Keywords:** Boston naming test, Alzheimer's disease, mild cognitive impairment, naming deficit, language impairment

## Abstract

Despite the ubiquity of the Boston naming test (BNT) in clinical practice and research, concerns have been expressed about its poor quality pictures, insufficient psychometric properties, and cultural bias in non-English language backgrounds. We modified the black-and-white BNT with a set of color pictures since color effects have been suggested to improve naming accuracy in the visual naming test. This study aimed to examine and compare the reliability and validity of the color-picture version of BNT (CP-BNT) and the black-and-white version of BNT (BW-BNT) to differentiate amnestic mild cognitive impairment (aMCI) or mild Alzheimer's disease (AD) from the cognitive normals. This study included two subgroups, and each subgroup had 101 normal controls, 51 aMCI, and 52 mild AD. One subgroup undertook BW-BNT and the other conducted CP-BNT. The reliability, convergent and discriminant validity, and the diagnostic accuracy of two versions of BNT were evaluated. The CP-BNT showed a greater area under the curve (AUC) than the BW-BNT for aMCI (80.3 vs.s 69.4%) and mild AD (93.5 vs. 77.6%). The CP-BNT also demonstrated better convergent validity with CDR global scores and better reliability (Cronbach's coefficient 0.66 for the CP-BNT vs. 0.55 for the BW-BNT). At the optimal cutoff value of spontaneous naming, the CP-BNT demonstrated improved sensitivity and specificity for differentiating mild AD from NC with a higher positive predictive value, negative predictive value, and lower false-positive rate. Compared with BW-BNT, CP-BNT is a more reliable and valid test to assess cognitive and naming impairment.

## Introduction

Naming difficulty is the most common symptom of language dysfunction seen in neurological diseases such as Alzheimer's disease, frontotemporal dementia, stroke, encephalitis, temporal lobe epilepsy, brain trauma, etc. (1). Language neuroscience research suggests that visual confrontation naming relies on specific and distributed brain networks that operate on sequential cognitive processes, spanning from visual recognition, semantic activation, lexical retrieval, and articulation of word form. Given the complexity of visual naming processes, damage in several cortical and/or subcortical regions would result in naming impairment (1). As a result, naming impairment could be used as a marker of clinical feature and severity of disease, as well as the predictor of stability of neurological disease (2).

The Boston naming test (BNT) is the most commonly used neuropsychological instrument for visual confrontation naming (3, 4). In the prodromal stage of AD, BNT was regarded as a valuable tool to characterize disease severity and track clinical progression. Longitudinal studies supported that naming impairment provided evidence for the clinical stability and diagnostic reliability of amnestic mild cognitive impairment (2, 5). Individuals with amnestic MCI who had naming deficits had more than twice the risk of converting to dementia than those who had single-domain amnestic MCI (2).

Despite the widespread popularity of the BNT in clinical practice and research, there has been a longstanding controversy about BNT. These arguments are that the BNT has inadequate sensitivity to detect subtle naming impairment, out-of-date line drawings, problematic psychometric properties, and cultural bias, especially used in Non-English language backgrounds (6, 7). For example, the 30-item BNT had an acceptable sensitivity in a sample of patients with mild AD, but poor sensitivity when applied in a clinical setting of MCI (AUC = 0.87; 61% sensitivity and 89% specificity) (8). Studies suggested some items of BNT had low difficulty and weak ability to discriminate between persons with low vs. high naming ability (9). This means that the BNT needs some modifications to improve its psychological properties.

The Boston naming test has also been criticized for the poor quality of its black-and-white line drawings (6). In Chinese clinical application, both examiners and examinees often complain that even normal subjects may have difficulties recognizing some original items of BNT. Since the BW-BNT was developed in 1983 and stayed largely unchanged in its format since then, many line drawings in the original version differed obviously from the target objects in the current time (3, 4, 10). There may be possible cohort effects as well as cultural bias that make the black-and-white line drawings liable to be misperceived in non-English language backgrounds. For example, the drawing of a “pretzel” is often misperceived as a snake (6). The “igloo” is usually mistaken for a mud oven due to its resemblance in some parts of South America (11). From an ecological view, the diagnostic validity of studies using black-and-white line drawings has been questioned (12). Consequently, the number of naming tests using color stimuli, which provided a more realistic representation of objects, has been progressively increasing (13–15). A recent meta-analysis of 35 studies suggested that color information had a color effect on object recognition to improve naming accuracy and speed correct response times (16).

The 30-item Chinese version of BNT has been adapted several times since the 1990s. Given that Chinese is a logographic language, phonemic cueing is not applicable to this population. The adaptation in 2004 developed a new word choice cuing paradigm to replace the phonemic cue (17). The reliability and validity of the 30-item Chinese version of BNT have been validated (18, 19), but the application of BNT is still limited by its poor psychometric properties and cultural bias. For the need for greater sensitivity and cultural appropriateness in the neuropsychological assessment, we modified the Chinese version of the 30-item BNT with a set of color pictures to replace the original black-and-white line drawings. The study aims to determine and compare the reliability, convergent and discriminant validity, and diagnostic accuracy of CP-BNT and BW-BNT in a Chinese sample with cognitive normals, mild cognitive impairment patients due to Alzheimer's disease, and mild Alzheimer's disease.

## Method

### Participants and Diagnosis

All participants (aged 55–85 years) included normal cognitive control, patients with amnestic mild cognitive impairment (aMCI), and mild Alzheimer's disease (mild AD). Patients were consecutively recruited from the memory and language clinic, Department of Neurology, Xuanwu Hospital, Capital Medical University, Beijing from January 2015 to December 2018. The normal controls were volunteers from the community and the spouses or caregivers of patients. They were community-dwelling, cognitive, and neurologically healthy individuals.

A guideline for referral to neuropsychological assessment was the MMSE score ≥20 at the initial visit to the clinic, but patients with lower MMSE scores may also be referred if deemed relevant (e.g., in the case of low education). All participants had an extensive diagnostic assessment including a clinical interview, neurological and physical examination, a comprehensive neuropsychological assessment battery published previously (20), routine laboratory examinations (comprising vitamin B12 and folate dosage, serology for syphilis, and thyroid hormones), and brain structural scans (MRI and/or CT). Subjects were excluded from the study if any of the following conditions applied: (1) evidence of stroke or Fazekas score ≥2, as determined by neuroradiological or clinical examination; (2) history of severe head injury; (3) current psychiatric diagnosis; (4) any medical condition that leads to severe cognitive deterioration, including thyroid dysfunction, diabetes, renal, respiratory, cardiac, and hepatic disease; (5) present or past abuse or daily use of alcohol or drugs. After completion of the diagnostic workup, the multidisciplinary staff established a consensus diagnostic classification.

An aMCI patient was diagnosed according to the clinical core criteria of MCI due to AD proposed by the National Institute on Aging-Alzheimer's Association workgroups (NIA-AA) (21). The criteria included: (1) memory impairment with an insidious onset and gradual progression; (2) an objective memory impairment was defined by impaired performance on the Chinese version of WHO/UCLA-AVLT delayed recall below the cut-off value (≤9) (20); (3) score higher than the cutoff points for dementia in MMSE, >26 for middle school and above; (4) clinical dementia rating (CDR) of 0.5, with a score of at least 0.5 on the memory box; (5) ability preserved to perform daily activities and social functions; (6) neuroimaging features consistent with incipient AD (i.e., hippocampus and entorhinal cortex atrophy) and no other lesions; and (7) no other medical or neuropsychiatric conditions that could account for the cognitive impairment. AD dementia was diagnosed according to the clinical core criteria of AD dementia proposed by NIA-AA, and only participants with mild AD dementia with a global CDR score of 1 were included (22).

These criteria resulted in 408 participants, including 202 cognitively normal controls, 102 participants with aMCI, and 104 with mild AD. All participants were randomly divided into two subgroups by cognitive level. Each subgroup had 101 NCs, 51 patients with aMCI, and 52 patients with mild AD. One subgroup undertook the black-and-white line drawing version of BNT, and the other subgroup performed the new color-picture version of BNT. Written informed consent was obtained from all participants and/or their families and approved by the ethical committee of Xuanwu Hospital.

### The Development of the Color-Picture Version of BNT (CP-BNT)

The present study adopted the same items from the Chinese version of BNT published in 2004 (17, 19). Thirty color pictures were used to replace the black-and-white line drawings. Twenty-nine pictures were photographs of real objects. Only the picture for the item “sea horse” was a color drawing obtained from the internet. Supplementary Figure 1 demonstrated the pictures of the item “broom” in BW-BNT and CP-BNT. The change in picture format did not change the target response word. As a result, the CP-BNT did not change the word frequency and familiarity, the number and length of syllables, and difficulty of articulation for the 30 items. All the color pictures were placed on a plain white or plain colored background, and the mean dimension of the images was 265 x 223 pixels. The CP-BNT was displayed on 19-inch LCD color monitors with a screen resolution of 128 x 800 pixels and a 64-bit color mode.

### The Administration of the CP-BNT and the BW-BNT

All participants were administered all 30 items of the CP- BNT or the BW-BNT, starting from item 1, with no setting of a basal or discontinuation rule. Each participant was asked to name the picture with a single word as precisely as possible once it appeared on the computer screen (CP-BNT) or the paper card (BW-BNT). If the participant named the item correctly, the examiner proceeded to the next item. If the participant gave a wrong response or gave no response within 20 s, then a semantic cue was given. If the participant could not name the object correctly with the semantic cue, the three-choice recognition task was given, and the choice of the examinee was recorded.

The measures of naming performance included the total number of correct items on spontaneous naming, semantic cuing, and word recognition. Scores of spontaneous naming (SN) were computed as the number of items correctly named spontaneously, ranging from 0 to 30. The score of semantic cueing (SC) is the number of correct responses after giving the semantic cue, as well as the score of word recognition (WR), ranging from 0 to 30. Two additional descriptive scores, the percentage of correct responses on semantic cuing or word recognition (SC and %WR), were calculated for all the participants by dividing the number of correct responses during semantic cuing or recognition by the number of errors preceding the cues, ranging from 0 to 100%. The total score of BNT is the sum of the scores of SN, SC, and WR, ranging from 0 to 30.

### Statistical Analyses

The Chi-square and Mann–Whitney U tests were used to examine the differences in demographic characteristics, clinical cognitive function, and performance of two versions of BNT between the two subgroups by the same cognitive level (i.e., normal control, aMCI, and mild AD, respectively). Within each subgroup, the Kruskal–Wallis test was used to examine the differences in demographic characteristics, cognitive function, and BNT performance among the three cognitive levels. The convergent validity of two versions of BNT was assessed by both univariable and multivariable regression analysis for the association between SN score and demographic and cognitive factors. For each subgroup, the area under the curve (AUC) with SN raw score was identified with receiver operating characteristics (ROC) curve analysis to differentiate aMCI or mild AD from NC. The SN optimal cut-off score was calculated based on Youden's J index (sensitivity + specificity −1). We also evaluated sensitivity and specificity, false-positive rate (FPR), positive predictive value (PPV), negative predictive value (NPV), and Likelihood ratio for a positive test result (LR+) at the optimal SN cut-off value for aMCI and mild AD in the two subgroups.

To adjust for the effect of demographic variables (age, sex, and education) on the diagnostic accuracy, the T-score of SN was calculated based on the normal control demographically in the two subgroups, respectively. A ROC analysis with an adjusted T-SN score was conducted for aMCI and mild AD in the two subgroups.

The reliabilities of the two versions were examined using Cronbach's alpha coefficients, respectively. We considered *p* < 0.05 (two-sided) as the statistical significance. Statistical analyses were performed using SPSS Statistics version 22.0 (IBM Corp., Armonk, NY) for Windows.

## Results

### Demographic Characteristics and Clinical Cognitive Function

Table 1 shows the demographic characteristics and clinical cognitive function of the two subgroups (six diagnostic groups). There was no significant difference in the demographic characteristics between the two subgroups based on cognitive levels. Compared with subgroup 1, NC and aMCI patients in subgroup 2 had significantly higher MMSE and MoCA scores. There was no statistically significant difference in MMSE and MoCA scores between patients with mild AD in the two subgroups.

**Table 1:** Demographic, cognitive characteristics, and performance of BNT in the two subgroups.

	**Subgroup 1 with BW-BNT**			**Subgroup 2 with CP-BNT**		
	**NC (*n* = 101)**	**aMCI (*n* = 51)**	**Mild AD (*n* = 52)**	**NC (*n* = 101)**	**aMCI (*n* = 51)**	**Mild AD (*n* = 52)**
Male (%)	48 (47.5%)	27 (52.9%)	31 (59.6%)	47 (46.5%)	21 (41.2%)	22 (42.3%)
Age (ys)	65.6 (5.9)	67.2 (6.7)	67.9 (7.2)^b^^*^	66.1 (6.0)	69.0 (5.6)	69.5 (7.1) ^b^^**^
Education (ys)	12.7 (2.6)	12.3 (2.8)	12.2 (2.7)	13.0 (2.7)	11.3 (3.2)	11.8 (3.5) ^b^^**^
MMSE	28.5 (1.1)^a^^**^	26.2 (1.4) ^a^^**^	21.4 (2.2) ^b^^**^	29.1 (1.2)	26.4 (2.1)	20.7 (2.4) ^b^^**^
MoCA	26.0 (1.5) ^a^^**^	20.2 (1.9) ^a^^**^	15.9 (2.3) ^b^^**^	26.6 (1.6)	21.8 (2.7)	16.2 (2.9) ^b^^**^
CDR	0 (0)	0.5 (0)	1 (0)	0 (0)	0.5(0)	1 (0)
Spontaneous naming	25.3 (2.8) ^a^^**^	23.1 (3.2) ^a^^**^	22.0 (3.2) ^b^^**^	27.6 (1.5)	24.6 (3.0)	23.0 (2.9) ^b^^**^
Semantic cueing	1.0 (1.1) ^a^^**^	1.3 (1.4)	1.1 (1.6)	0.4 (1.6)	0.6 (0.8)	0.8 (0.9) ^b^^**^
%Semantic cue	22.0 (24.9) ^a^^**^	15.9 (19.8)	15.4 (19.0)	13.3 (25.3)	12.9 (20.3)	12.4 (14.8)
Word recognition	3.1 (2.3) ^a^^**^	4.4 (2.5)	5.1 (2.9) ^b^^**^	1.9 (1.5)	4.3 (2.8)	5.1 (2.8) ^b^^**^
%Word recognition	78.3 (32.0) ^a^^*^	75.4 (25.7) ^a^^*^	72.4 (25.5) ^b^^*^	80.0 (37.6)	82.2 (27.9)	81.0 (22.5) ^b^^*^
Total score of BNT	29.4 (0.9) ^a^^**^	28.7 (1.3) ^a^^**^	28.2 (1.7) ^a^^**^^,^^b^^**^	29.9 (0.4)	29.5 (0.6)	28.9 (1.2) ^b^^**^

*Data are presented as mean (SDs) or number (proportion %)*.

*BW-BNT, Black-and white version of Boston naming test; CP-BNT, Color-picture version of Boston naming test; NC, Normal control; AD, Alzheimer's disease; aMCI, amnestic mild cognitive impairment; MMSE, mini-mental status examination; MoCA, Montreal cognitive assessment; CDR, Clinical dementia rating*.

a *Significantly different from cognitive matched groups*.

b *Significantly different within the BW-BNT or CP-BNT*.

* *p < 0.05*;

** *p < 0.01*.

When compared within subgroups, normal controls were significantly younger than patients with aMCI and mild AD (*p* = 0.026 for subgroup 1, *p* = 0.002 for subgroup 2). In subgroup 2, normal controls had significantly higher education than patients with aMCI and mild AD (*p* = 0.006).

### Differences in Performance Between the BW-BNT and the CP-BNT

As shown in Table 1, compared with subgroup 1 with BW-BNT, normal controls in subgroup 2 with CP-BNT scored significantly higher in SN (*p* < 0.01), %WR (*p* < 0.05), and total BNT (*p* < 0.01) with a significant decrease in SC, %SC, and WR. At the MCI level, patients with CP-BNT maintained significantly higher scores in SN (*p* < 0.01), %WR (*p* < 0.05), and total BNT (*p* < 0.01). However, at the mild dementia level, patients with CP-BNT only scored higher significantly in total BNT (*p* < 0.01). The differences in SN, SC, %SC, WR, and %WR were not significant.

### Results of Regression Analysis (Convergent Validity)

In subgroup 1 with BW-BNT, the univariable regression analyses showed that SN was significantly associated with gender, education, the MoCA, and CDR global score. Multiple stepwise regressions, however, revealed that only the MoCA (β = 0.45, *p* < 0.001) and gender (β = −0.19, *p* = 0.002) significantly predicted the SN score.

In the CP-BNT subgroup, the univariable regression analyses showed that SN was significantly associated with gender, education, the MMSE, MOCA, and CDR global score. However, using the multiple stepwise regressions, age (β = −0.21, *p* < 0.01), education (β = 0.17, *p* < 0.01), the MoCA (β = 0.27, *p* = 0.018), and CDR (β = −0.33, *p* < 0.01) significantly predicted the SN score. The results of univariable regression analyses are shown in the Supplementary Tables 1, 2.

### Results of Receiver Operating Characteristic Curve With SN Raw Score

**Figure 1:**
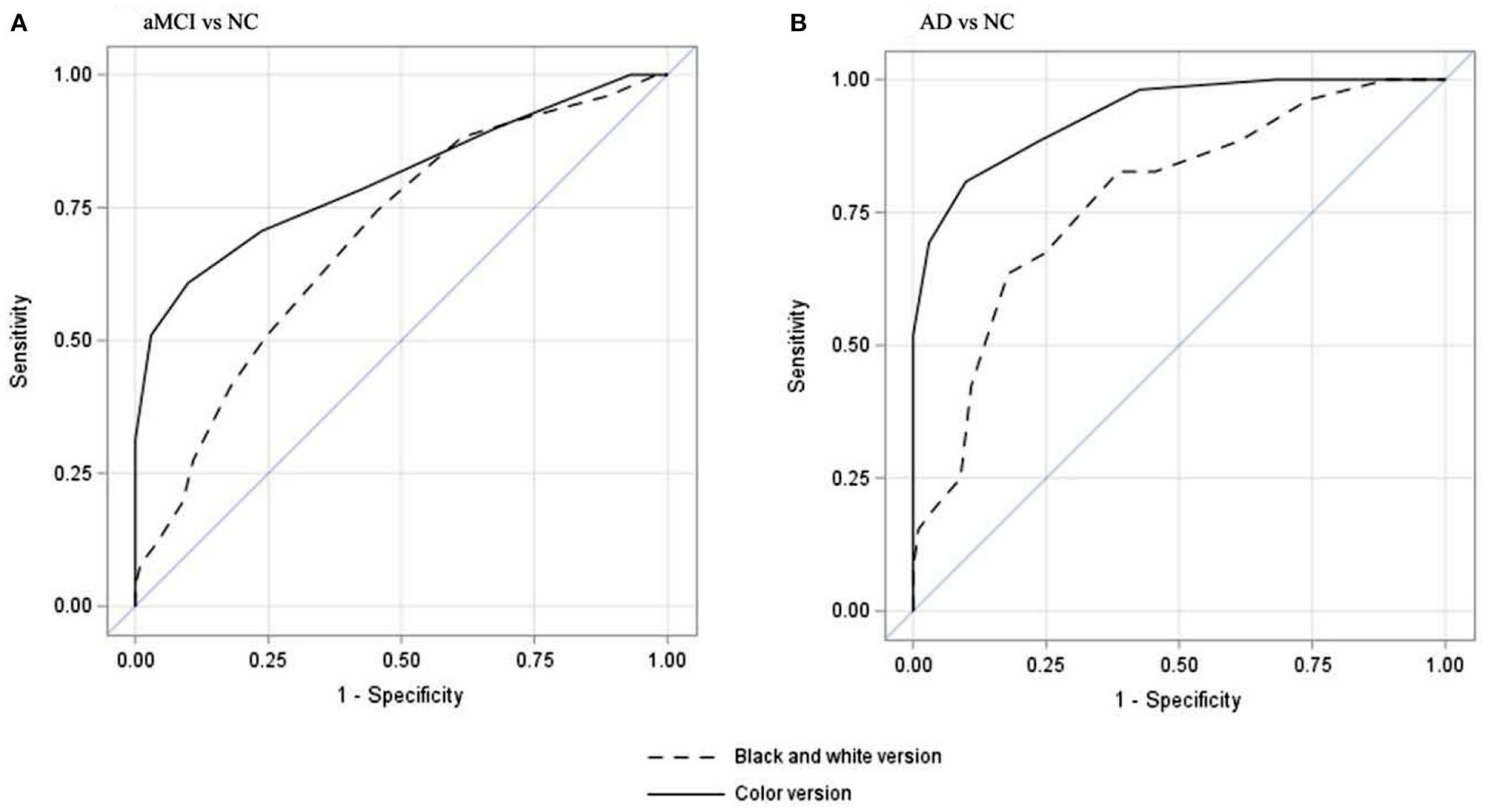
Receiver operating characteristic curves of spontaneous naming score to differentiate aMCI from NC **(A)** and to differentiate mild AD from NC **(B)**. **(A)** showed that CP-BNT had a better AUC (80.3%, 95%CI: 72.4–88.4%) than that of BW-BNT (AUC = 69.4%, 95%CI: 60.7–78.1%) to differentiate aMCI from NC. **(B)** showed that CP-BNT had a better AUC (93.5%, 95%CI: 89.6–97.2%) than that of BW-BNT (AUC =77.6% (95%CI: 69.9–85.3%) to differentiate AD from NC.

Figure 1 shows the area under the curves for aMCI and AD for the two versions of BNT with SN raw score. For both aMCI and mild AD, CP-BNT demonstrates greater AUC than BW-BNT.

As shown in Table 2, for CP-BNT, the optimal SN cut-off score of 25/26 yielded a sensitivity of 60.8% and a specificity of 90.1% for aMCI vs. NC with an AUC of 80.3% (95%CI: 72.4–88.4%), and a sensitivity of 80.8% and a specificity of 90.1% for mild AD with an AUC of 93.5% (95%CI: 89.6–97.2%).

**Table 2:** The diagnostic and differentiating ability of CP-BNT and BW-BNT.

**BNT version**	**Cut-off value**	**Sensitivity**	**Specificity**	**AUC (95%CI)**	**FPR**	**PPV**	**NPV**	**LR+**
**CP-BNT**								
aMCI vs. NC	25/26	60.8%	90.1%	80.3% (72.4; 88.4)	9.9%	75.6%	82.0%	6.1
AD vs. NC	25/26	80.8%	90.1%	93.5% (89.6; 97.2)	9.9%	80.8%	90.1%	8.2
**BW-BNT**								
aMCI vs. NC	25/26	74.5%	54.5%	69.4% (60.7; 78.1)	45.5%	45.2%	80.9%	1.6
AD vs. NC	22/23	63.5%	82.2%	77.6% (69.9; 85.3)	17.8%	64.7%	81.4%	3.6

*CP-BNT, the color picture version of Boston naming test; BW-BNT, the black-and-white version of Boston naming test; aMCI, amnestic mild cognitive impairment; NC, normal control; AD, Alzheimer's disease; AUC, Area under the ROC curve; CI, Confidence interval of the AUC; TPR, true positive rate; FPR, false positive rate; PPV, positive predictive value; NPV, negative predictive value; LR+, Likelihood ratio for a positive test result*.

For the BW-BNT, the optimal SN cut-off score of 25/26 yielded a sensitivity of 74.5% and a specificity of 54.5% for differentiating aMCI from NC with an AUC of 69.4% (95%CI: 60.7–78.1%). At the optimal cutoff of 22/23, the SN raw score differentiated NC and mild AD with a sensitivity of 63.5%, specificity of 82.2%, and an AUC of 77.6% (95%CI: 69.9–85.3%). Table 2 also shows that the CP-BNT outperformed the BW-BNT in PPV, NPV, and LR+, both for aMCI and mild AD. Also notably, the CP-BNT had much lower False positive rate (FPR) than the BW-BNT for aMCI (9.9 vs. 45.5%) and mild AD (9.9 vs. 17.8%).

### The ROC Analysis With the Adjusted SN Score

The AUC with adjusted SN scores for aMCI was 85.5% (95%CI: 79.4–91.7%) with the CP-BNT and 71.2% (95%CI: 62.3–80.0%) with the BP-BNT. The AUC of the adjusted SN scores for mild AD was 91.4% (95%CI: 86.5–96.3%) with the CP-BNT and 81.4% (95%CI: 74.1–88.6%) with the BP-BNT. The results also supported that CP-BNT improved diagnostic accuracy for aMCI and mild AD in comparison with BW-BNT. To facilitate the clinical application of CP-BNT, we adopted the SN raw score instead of the adjusted SN score in the present study for further discussion.

### Reliabilities of the Two Versions of BNT

The CP- BNT had an acceptable Cronbach's coefficient (α = 0.66). Compared with the CP-BNT, the BW-BNT had a poor Cronbach's coefficient (α = 0.55).

## Discussion

In the present study, we modified the black-and-white version of the 30-item BNT with a set of color pictures. We examined and compared the psychometric properties of the CP-BNT with the BW-BNT in a Chinese sample of cognitive normals and patients with aMCI and mild AD. The results supported that the CP-BNT outperformed the BW-BNT for detecting aMCI and mild AD with higher reliability, validity, and diagnostic accuracy in the Chinese language background.

Color is represented in a structural representation system and forms an essential attribute of the conceptual knowledge of prototypical objects. According to previous studies, the presence of color and photographic detail assists the processing of visual confrontation naming in terms of improving naming accuracy and speeding the response time (15, 23). It is generally thought that color effects arise at both the visual processing level and the semantic level, where color not only facilitates the visual recognition of objects but also provides additional cues to activate its semantic knowledge of prototypical objects (16, 24). In the present study, CP-BNT also demonstrated the color effect with higher SN scores and total scores than that of BW-BNT, as well as better diagnostic accuracy for detecting aMCI and AD. Besides the color effect, the CP-BNT adopted target pictures that were common in the everyday life of the Chinese.

These adaptations improved the cultural appropriateness of BNT in Chinese language background, which might further reduce the possibility of misperception. Consequently, in the present study, the CP-BNT showed a significantly lower false-positive rate than the BW-BNT in aMCI (9.9 vs. 45.5%) and mild AD (9.9 vs. 17.8%).

However, the color effect gradually diminished as the cognitive impairment deteriorated from NC to MCI and then to mild dementia. The same results were reported by Adlington et al. that naming for elderly controls improved linearly with color information, while patients with AD showed no benefit from the addition of color (25). One possible explanation is the early and common visual association cortex atrophy reported in patients with AD, especially atrophy of the angular gyrus and posterior middle temporal gyrus, which are supposed to interact with the default mode network and connect the visual recognition process with the semantic representations in the anterior temporal lobe (26, 27). Furthermore, Harnish et al. reported that visual discrimination abilities of objects strongly predicted performance on both picture naming and semantic association abilities in AD, but lacked the same predictive value for the controls (27). It may be, therefore, the case that the CP-BNT showed better discriminant ability to differentiate aMCI and mild AD from NC than the BW-BNT in the present study. The results also added indirect evidence that the visual deficit plays a role in the naming impairment in AD.

Results of stepwise regression models suggested that the CP-BNT had better convergent validity with the CDR global score than the BW-BNT. Patients with mild AD tended to perform worse in CP-BNT than aMCI patients, which suggests CP-BNT might be used as a better marker for disease deterioration than BW-BNT. Moreover, CP-BNT was significantly associated with age and education, while BW-BNT was only related to gender. Since BNT is a cultural and language relevance test, a majority of studies have found that age and education level are particularly related to the performance of BNT (28). However, in the Chinese sample, the effect of age and education on BNT was still controversial (18, 19). The discrepancies could be attributed to varying research methods and participant characteristics (e.g., age range, educational levels) (28). However, in the present study, the two subgroups had matched demographic characteristics and the same language background. The improvement in psychometric properties of BNT and better convergent validity was likely due to the changes in the picture format, which improved the picture quality and cultural appropriateness.

Collectively, our study offers methodological strengths. Although a few studies have supported that visual naming tests with color stimuli have better ecological validity than tests with black-and-white material (13–15), this is the first study to modify BNT with color pictures. It is also the third adaptation of the Chinese version of BNT to make it more culturally appropriate in the context of the Chinese language (17). The results supported empirically that CP-BNT outperformed the BW-BNT by overcoming some shortcomings such as low quality of pictures, poor psychometric properties, cultural bias, and thus improved the diagnostic accuracy for detecting aMCI and mild AD.

It is important to note that the current study has several limitations. First, our sample size was limited and from a clinic-based sample. The results of normal controls cannot be truly representative of the general population. Second, as the current sample was likely to be younger and well-educated, samples with limited education should be cautious to use the SN cutoff value of 25/26, which may result in an overestimation of the naming deficit. Third, the CP-BNT can be believed to have better diagnostic accuracy for detecting aMCI, although the sensitivity (60.8%) is still not optimal. The results also revealed that some items showed a marked ceiling effect. The value of this test lies in its ability to detect the severe to moderate level of naming deficit rather than its standalone ability to identify specific patients with aMCI (29). For the patients with single-domain amnestic mild cognitive impairment, episodic memory appears to be affected before other cognitive domains, although those individuals who manifest impairments in one or more cognitive domains are more likely to convert to dementia (2, 30).

In summary, the current study provided empirical evidence supporting that CP-BNT outperformed BW-BNT in validity and reliability to detect patients with aMCI and AD. The study deepened our understanding of the psychometric properties of BNT and improved the manipulability in Chinese language background. We suggest that the CP-BNT could be an ecological alternative to the original BNT, especially, but not exclusively in the Chinese language background.

## Data Availability Statement

The raw data supporting the conclusions of this article will be made available by the authors, without undue reservation.

## Ethics Statement

The studies involving human participants were reviewed and approved by the Ethical Committee of Xuanwu Hospital, CMU. The patients/participants provided their written informed consent to participate in this study.

## Author Contributions

DL and Y-YY: study concept and design. DL, Y-YY, LL, and FW: acquisition of clinical data. DL and LL: acquisition of neuropsychological data. NH: analysis of data and statistical analysis. DL, NH, LL, MZ, and FW: drafted or revised the manuscript. MZ: performed MR/CT brain scan and provided a neuroimaging diagnosis. L-MF and S-SR: contacted patients, family caregivers, and normal controls. DL: acquired financial support. All authors contributed to the article and approved the submitted version.

## Funding

This work was supported by the Natural Science Foundation of Beijing Municipality (No. L182049) and the National Key Research and Development Project of China (No. 2018YFC1315204).

## Conflict of Interest

The authors declare that the research was conducted in the absence of any commercial or financial relationships that could be construed as a potential conflict of interest.

## Publisher's Note

All claims expressed in this article are solely those of the authors and do not necessarily represent those of their affiliated organizations, or those of the publisher, the editors and the reviewers. Any product that may be evaluated in this article, or claim that may be made by its manufacturer, is not guaranteed or endorsed by the publisher.
