# Supplementary material for: A Color-Picture Version of Boston Naming Test Outperformed the Black-and-White Version in Discriminating Amnestic Mild Cognitive Impairment and Mild Alzheimer's Disease
Source: Front Neurol. 2022 Apr 25;13:884460. doi: 10.3389/fneur.2022.884460 (PMC9082938; doi:10.3389/fneur.2022.884460)
Supplement: Supplementary file 1 [file Table_1.docx]

Supplementary Material

# Supplementary Figure1


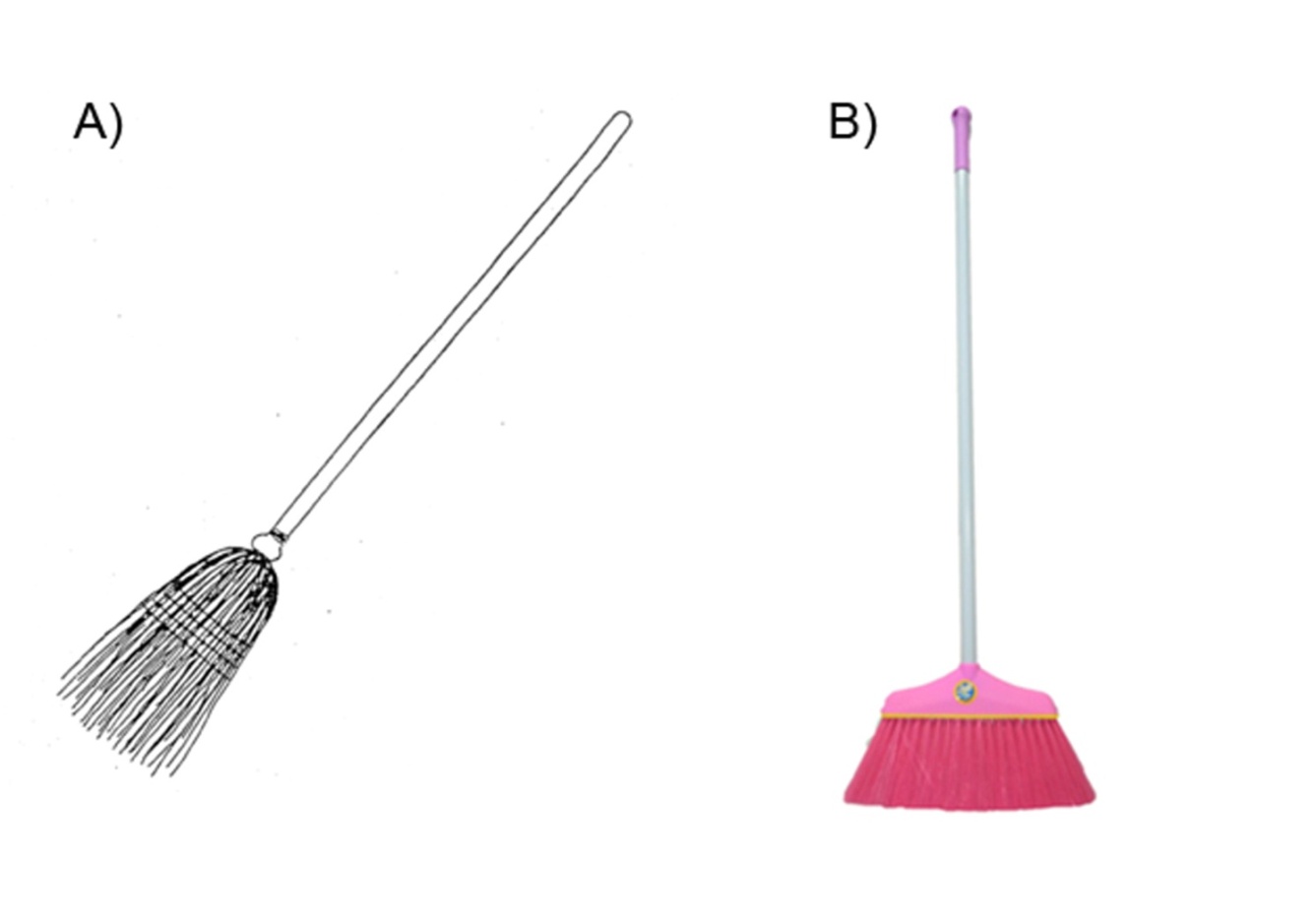


**eFig 1**  A) The black-and-white line draw of item broom in BW-BNT; B) The color picture of item broom in CP-BNT

# Supplementary eTable 1

eTable 1 The univariable regression analyses of BW-BNT (Spearman’s correlation)

| Variables | SN | Gender | Age | Education | MMSE | MoCA |
| --- | --- | --- | --- | --- | --- | --- |
| SN |  |  |  |  |  |  |
| Gender | -0.16* |  |  |  |  |  |
| Age | -0.03 | -0.14* |  |  |  |  |
| Education | 0.16* | -0.18** | 0.04 |  |  |  |
| MMSE | 0.39** | 0.12* | -0.18** | 0.02 |  |  |
| MoCA | 0.44** | 0.06 | -0.16* | -0.16* | 0.85** |  |
| CDR | -0.42** | -0.11 | 0.15* | -0.07 | -0.88** | -0.92** |

Note:

BW-BNT = Black-and white version of Boston naming test; SN= Spontaneous naming;

MMSE = mini-mental status examination; MoCA =Montreal cognitive assessment;

CDR = Clinical dementia rating scale

* *p*< .05; ** *p*< .01

# Supplementary eTable2

eTable 2 The univariable regression analyses of CP-BNT (Spearman’s correlation)

|  | SN | Gender | Age | Education | MMSE | MOCA |
| --- | --- | --- | --- | --- | --- | --- |
| SN |  |  |  |  |  |  |
| Gender | -0.13* |  |  |  |  |  |
| Age | -0.31** | 0.07 |  |  |  |  |
| Education | 0.23** | -0.08 | 0.21** |  |  |  |
| MMSE | 0.51** | -0.02 | -0.18** | 0.17** |  |  |
| MOCA | 0.63** | -0.05 | -0.18** | 0.22** | 0.90** |  |
| CDR | -0.64** | 0.05 | 0.24** | -0.17** | -0.87** | -0.88** |

Note:

CP-BNT = color-picture version of Boston naming test; SN= Spontaneous naming;

MMSE = mini-mental status examination; MoCA =Montreal cognitive assessment;

CDR = Clinical dementia rating scale

* *p*< .05; ** *p*< .01

# Supplementary eTable3

eTable 3The neuropsychological tests of two subgroups

|  | Subgroup1 | | | Subgroup1 | | |  |  |  |
| --- | --- | --- | --- | --- | --- | --- | --- | --- | --- |
| Measures | NC | aMCI | AD | NC | aMCI | AD | p1 | p2 | p3 |
| WHO/UCLA AVLT-I | 54.23±7.53 | 36.61±6.40 | 23.71±7.27 | 52.34±6.08 | 35.33±9.18 | 22.26±6.53 | 0.051 | 0.418 | 0.287 |
| WHO/UCLA AVLT-D | 12.51±2.03 | 5.43±3.07 | 0.86±1.68 | 12.17±1.72 | 4.22±3.50 | 1.04±1.67 | 0.205 | 0.065 | 0.6 |
| Digit span forward | 8.61±0.76 | 8.37±1.18 | 7.58±1.30 | 8.49±0.96 | 8.16±0.95 | 7.59±1.22 | 0.291 | 0.312 | 0.964 |
| Digit span backward | 5.90±1.35 | 5.10±1.39 | 3.81±1.21 | 5.49±1.25 | 5.04±1.08 | 3.86±1.17 | 0.028 | 0.812 | 0.814 |
| TMT part A | 38.22±10.28 | 51.15±17.90 | 71.47±37.87 | 40.17±11.12 | 47.41±15.72 | 105.48±61.61 | 0.2 | 0.264 | 0.001 |
| TMT part B | 62.00±20.97 | 95.74±56.21 | 215.42±91.77 | 62.44±21.52 | 102.04±58.63 | 213.52±90.71 | 0.884 | 0.581 | 0.916 |
| Rey complex figure copy | 35.58±0.65 | 34.80±1.15 | 25.81±9.59 | 35.55±0.75 | 35.36±1.01 | 21.78±13.68 | 0.765 | 0.01 | 0.087 |
| Category fluency(1 min, animal) | 20.04±4.33 | 16.29±2.76 | 11.81±3.56 | 20.42±4.60 | 16.63±3.79 | 10.67±4.21 | 0.563 | 0.613 | 0.14 |
